# Machine Learning as a Support for the Diagnosis of Type 2 Diabetes

**DOI:** 10.3390/ijms24076775

**Published:** 2023-04-05

**Authors:** Antonio Agliata, Deborah Giordano, Francesco Bardozzo, Salvatore Bottiglieri, Angelo Facchiano, Roberto Tagliaferri

**Affiliations:** 1 Dipartimento di Scienze Aziendali, Management and Innovation Systems, Università degli Studi di Salerno, 84084 Fisciano, Italy; 2 BC Soft, Centro Direzionale, Via Taddeo da Sessa Isola F10, 80143 Napoli, Italy; 3 National Research Council, Institute of Food Science, Via Roma 64, 83100 Avellino, Italy

**Keywords:** T2DM, neural network, artificial intelligence

## Abstract

Diabetes is a chronic, metabolic disease characterized by high blood sugar levels. Among the main types of diabetes, type 2 is the most common. Early diagnosis and treatment can prevent or delay the onset of complications. Previous studies examined the application of machine learning techniques for prediction of the pathology, and here an artificial neural network shows very promising results as a possible valuable aid in the management and prevention of diabetes. Additionally, its superior ability for long-term predictions makes it an ideal choice for this field of study. We utilized machine learning methods to uncover previously undiscovered associations between an individual’s health status and the development of type 2 diabetes, with the goal of accurately predicting its onset or determining the individual’s risk level. Our study employed a binary classifier, trained on scratch, to identify potential nonlinear relationships between the onset of type 2 diabetes and a set of parameters obtained from patient measurements. Three datasets were utilized, i.e., the National Center for Health Statistics’ (NHANES) biennial survey, MIMIC-III and MIMIC-IV. These datasets were then combined to create a single dataset with the same number of individuals with and without type 2 diabetes. Since the dataset was balanced, the primary evaluation metric for the model was accuracy. The outcomes of this study were encouraging, with the model achieving accuracy levels of up to 86% and a ROC AUC value of 0.934. Further investigation is needed to improve the reliability of the model by considering multiple measurements from the same patient over time.

## 1. Introduction

Diabetes is a chronic, metabolic disorder characterized by high blood sugar levels, determined by insufficient production or function of insulin, a hormone produced by the pancreas, which regulates the uptake and metabolism of glucose, the main source of energy for the body’s cells.

This pathology can be classified into three specific categories: type 1 diabetes (T1DM), type 2 diabetes (T2DM), and gestational diabetes mellitus (GDM), related to different causes. In T1DM, also known as juvenile diabetes or insulin-dependent diabetes, an autoimmune mechanism destroys the insulin-producing cells in the pancreas with a complete lack of insulin production. In T2DM, the most common form and often associated with obesity and a sedentary lifestyle, multifactorial causes (such as genetic and environmental factors) induce resistance to insulin action, and the pancreas is unable to produce enough insulin to balance this resistance. GDM is often diagnosed in the second/third trimester of pregnancy in women not affected before gestation. In the last category instead belong patients whose diabetes is drug- or chemical-induced or resulting from other pathologies such as disease of exocrine pancreas or monogenetic syndromes (i.e., neonatal diabetes and maturity-onset diabetes of the young). T1DM and T2DM are heterogeneous diseases, often not easy to categorize in patients, which manifests clinically as hyperglycemia. Once hyperglycemia occurs, people with all forms of diabetes are at risk for developing the same chronic complications, such as kidney disease, heart disease, stroke, nerve damage, and vision loss, although rates of progression may differ [1]. Early diagnosis and treatment, including lifestyle changes and medication, can prevent or delay the onset of complications, in particular in T2DM.

Diabetes is one of the top ten causes of death worldwide. According to the 10th of the IDF Diabetes Atlas, the global diabetes prevalence in 20–79 year-olds in 2021 was evaluated to be 10.5% (536.6 million people), and projections estimate it rising to 12.2% (783.2 million) in 2045 [2]. Nowadays, the management of diabetes still represents a challenge because, despite the 11.5% of total global health expenditure spent on diabetes, almost one in two adults suffering from this pathology remain unaware of their status [3].

Artificial intelligence (AI) applies computer science and technology to problem-solving based on large data sets. It is a fast-growing field, which found many applications in biology and medicine research, as demonstrated in a large body of scientific literature [4,5,6,7], and also in studies on diabetes, not only in the therapeutic monitoring but also in the prediction of new-onset diabetes, and of future complications related to this pathology, and it is estimated that this methodology will help in bringing down the diabetes global prevalence of 8.8% [8].

Among AI techniques, machine learning (ML) and deep learning (DL) models are widely used. In particular, supervised ML is defined as when a system is trained using a database consisting of decoded reference examples and models (already equipped with all useful attributes that can help the learning system to catalog and classify the examples correctly). In this way, the ML algorithms will be able to analyze the data more precisely and solve problems or tasks automatically, based on previous experience and the provided examples indicated as ‘appropriate’. A supervised learning algorithm can produce an inductive hypothesis, i.e., a resolution model for general problems, starting from a set of particular problems. DL is based on artificial neural networks, constituted by nodes (or neurons), i.e., the fundamental computational component, organized in layers: an input layer consists of one input node for each single input feature, and receives the raw input data; one or more hidden layers perform computations on the input signals received from the previous layer, applying a weighted sum of its inputs, adds a bias term, with weights and biases learned during the training process to optimize the network’s performance, and applies an activation function to produce an output signal that is sent to the next layer of the network; the latter is the output layer that produces the final output of the network, which can be in the form of a classification label, a regression value, a probability distribution, or any other type of output that the network is designed to produce.

These approaches have been employed to create noninvasive diabetes risk forecasting models by the analysis of morphological features such as tongue [9] or retinal fundus images [10], or from special patterns of body fat distribution exploiting imaging from abdominal computed tomography [11] or magnetic resonance [12]. In the last case, models were trained for insulin sensitivity, glycated hemoglobin A1c (HbA1c), age, sex, Body Mass Index (BMI), prediabetes, and the occurrence of diabetes, reaching an AUC at 87% for T2DM discernment and 68% for prediabetes. Several studies demonstrate that ML could be a promising tool to maximize new-onset diabetes prediction than conventional statistics models, reporting an accuracy variable from 71% to 94% and exploiting a dataset composed of a minimum of 3700 patients up to a maximum of 2 million [13]. In particular, Ravault and colleagues [14] applied an ML approach to routinely collected health administrative data of over 2 million general population with a DM prevalence of just 1% and examined more than 300 features derived from demographic details, geographic information, chronic conditions, and health care use history. This method resulted in being able to detect new-onset DM within 5 years with the performance of AUC 0.8026.

ML and DL applications are also employed for managing T2MD and its evolutions; for example, a personalized postprandial-targeting diet, relying on an ML algorithm that integrates clinical and microbiome features, was used to predict personal postprandial glucose response, in order to control glycemic and metabolic health in patients with newly diagnosed T2DM [15]. Again, a stepwise approach, based on the combination of machine learning methods, probability graph models, classical statistical modeling tools, and in-house algorithm, was proposed to select drug combinations for compensating carbohydrate metabolism for T2DM patients [16]. ML-based predictors derived from baseline HbA1c level, comorbidities, demographic variables, and baseline metformin dosage were exploited for predicting the achievement and also for maintaining HbA1c < 7.0% after one year of metformin treatment [17]. Moreover, a device that uses convolutional neural networks trained to interpret retinal appearance [18] has been authorized by FDA to follow-up with patients with diabetes for the development of diabetic retinopathy and a mobile app, trained to interpret images of feet [19], has been developed in order to monitor diabetic foot pathology.

However, despite the huge advances of AI in T2DM, feature selection and dataset composition remain a tricky point to deal with. The analysis of diabetes data is complicated because most of the relevant data are nonlinear, non-normal, and correlation structure, leading to a paucity of supporting data to build logical and accurate algorithms. Furthermore, for this disease, huge data sets are generated just due to the heterogeneous nature and chronic course of the pathology [8]. Therefore, to overcome this difficulty, various ML and DL algorithms have been developed, and it is a common belief that the use of large amounts of organized data will dramatically improve the predictive accuracy of disease diagnosis, prevention, and treatment in diabetes [13]. Actually, for almost all the applications previously cited, prediction models are combined, used in various datasets for patient condition evaluation, and trained on features of a heterogeneous and large cohort of patients to enhance the feasibility of prognosticating factors. This underlines that ML and DL algorithms are promising approaches for controlling blood glucose and diabetes; however, they should be improved and employed in large datasets to affirm their applicability [20].

Feature selection, as already mentioned, is not a trivial point; the choice depends on the typology of the predictor that the expert wants to realize but also on data availability. There is no agreement on the specific features to create a predictive model for T2DM. Sometimes taking into account a large number of features may result in greater efficiency of the predictor, but often the accuracy decreases significantly when the dataset is too large and complex [21]. Moreover, the larger the amount of data selected, the more difficult their collection over time. Often, some data arise from expensive and/or invasive analysis not applicable for follow-up screening of all the patients involved in the dataset, thus the risk is losing data over time. In other cases, instead, not all the features selected turn out to be relevant for the accuracy of the predictor, demographic features, and insulin, for example, did not add any performance improvement for diabetes forecasting [22]. Moreover, diabetes risk factors and their related features are really a lot, and often their true correlation with T2DM is still debated [23].

In the present study, to create an accurate T2DM predictor model, we decided to choose a limited set of features that do not require excessive questioning or testing of patients, are easy to collect also in an extended period, and for which literature studies that correlated them to the pathology of interest are available. We decided to use, for the first time, data composed of suitable features collected from three different datasets: the National Health and Nutrition Examination Survey (NHANES) of the National Center for Health Statistics biennial survey [24], the MIMIC-III [25], and the MIMIC-IV [26] datasets, which contain clinical data of patients from the Beth Israel Deaconess Medical Center. A balanced dataset has been created by using these datasets, and a binary classifier has been developed.

## 2. Results

### 2.1. Dataset Statistics

Data retrieved from three datasets were merged and preprocessed (see Section 4) to remove features not of interest and implausible values, and to obtain a balanced dataset for the analysis. We report in Table 1 and Table 2 the statistics of the data, before and after the preprocessing phase, respectively. The columns identify the number of occurrences (counts) of non-zero values (i.e., numeric values that are not equal to zero), the mean, the standard deviation, the minimum value, and the maximum value.

**Figure 1:**
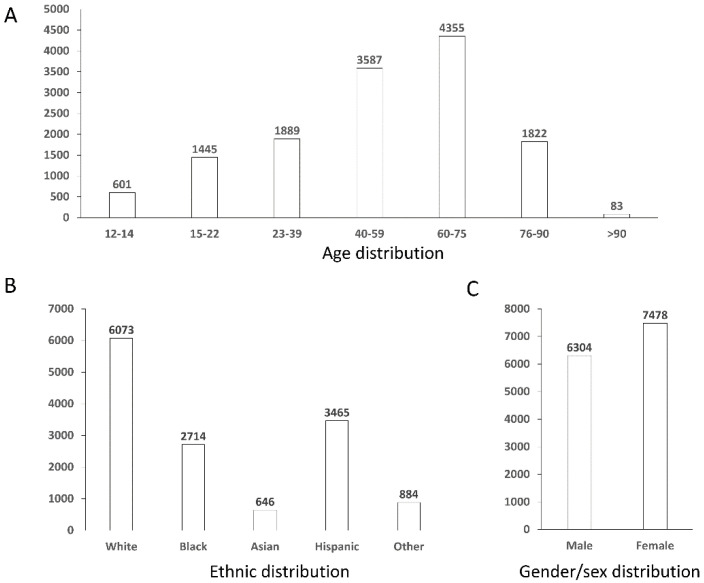
Distribution graphs for the population of the final dataset. Panel (**A**) reports distribution by age ranges, panel (**B**) by ethnic groups, and panel (**C**) by gender/sex.

After the preprocessing phase, a final data set of 13,687 rows has been obtained, with a balanced number of non-diabetics and diabetics individuals. Figure 1 shows the distribution graphs for the population of the final dataset, broken down by age ranges, gender/sex, and ethnicity.

### 2.2. Hyperparameters’ Tuning

To determine the optimal number of nodes in the hidden layer, a grid search approach was employed. The minimum and maximum number of nodes considered were five and fifteen, respectively. As a result, ten experiments were conducted, one for each number of nodes considered. For each experiment, a model with the following characteristics was created:

- Learning rate: 0.001;
- Loss Function: Binary cross-entropy;
- Optimization algorithm: Stochastic Gradient Descent;
- Trigger function for hidden layer: ReLU;
- Trigger function for the output layer: sigmoids;
- Number of nodes in the hidden layer: x ∈ [5,15].

The model was trained for a hundred epochs to evaluate the accuracy value attained with that configuration of nodes on a validation set, which was previously extracted from the training set, with a size of 20% of the total. All experiments were repeated ten times, and the average accuracy values were calculated. Table 3 displays the average accuracy values for each experiment, arranged in descending order (the higher the best).

Experiments were conducted to identify the optimizer that provided the best performance. Four optimization algorithms were considered: Stochastic Gradient Descent (SGD), Adaptive Moment Estimation (ADAM) [27], Root Mean Squared Propagation (RMSPROP) [28] and Levenberg–Marquardt (LM) [29].

In this case, k-fold cross-validation was employed as the validation technique, with k equal to 9. The experiments were repeated multiple times, for a total of 11 repetitions. Table 4 presents the average accuracy obtained for each optimizer for each fold in each of the eleven experiments along with the standard deviation, illustrating that ADAM is the most efficient algorithm.

### 2.3. Model Ensemble

For each optimizer, it was decided to utilize an ensemble of the 9 × 11 models obtained in the previous step. A voting scheme was employed, in which all models in the ensemble returned a result, and the outcome returned by the majority of models was then returned as the final prediction. The ensemble was initially trained on the training set and then evaluated for performance on the test set. The results are shown in Table 5.

### 2.4. Feature Reduction

**Figure 2:**
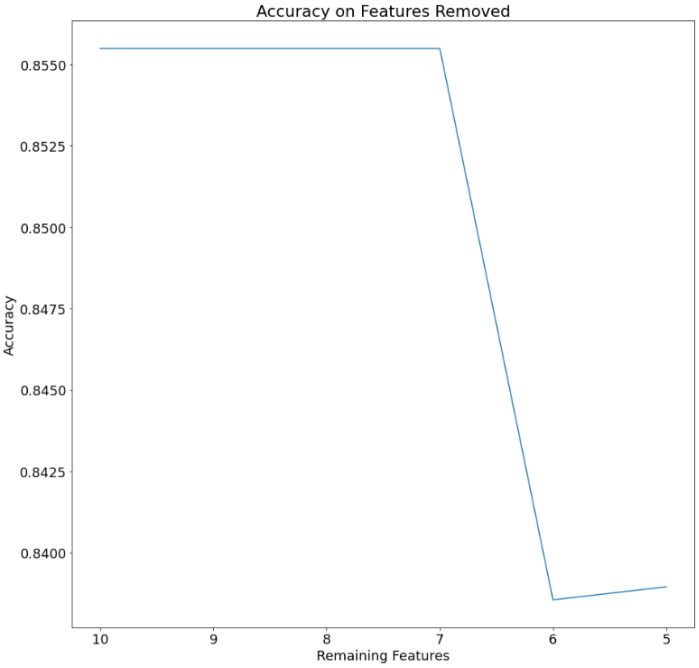
Accuracy of the model by sequentially eliminating 1 feature at each time.

A feature reduction approach was also attempted; one feature was sequentially eliminated at each step (the less significant ones on the validation set), and the model was retrained using the ADAM optimizer on the training set. This procedure was repeated thirty times, and the average accuracy obtained from each iteration was calculated. The features removed one at each time were in the order triglycerides, age, Body Mass Index, and Systolic Blood Pressure. The performance of the models on the test set was finally evaluated. The graph in Figure 2 illustrates the extent of model performance degradation as a result of the feature reduction. Feature reduction was based on accuracy. When the less significant features were eliminated one at a time, the accuracy did not change until six features remained.

### 2.5. Validation

**Figure 3:**
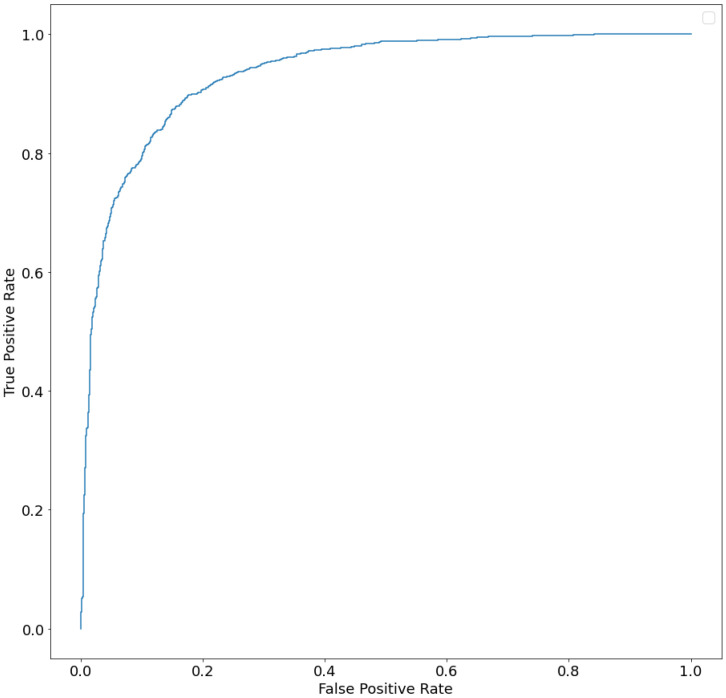
ROC curve (AUC) of ensemble model.

For the validation of the best model obtained (see Section 4.5 for methodology details), the ROC (Receiver Operating Characteristic) and the corresponding ROC AUC (Area Under Curve) score were calculated. The graph with the relevant ROC curve is shown in Figure 3. The calculated ROC AUC value is 0.934.

### 2.6. Calibration

**Figure 4:**
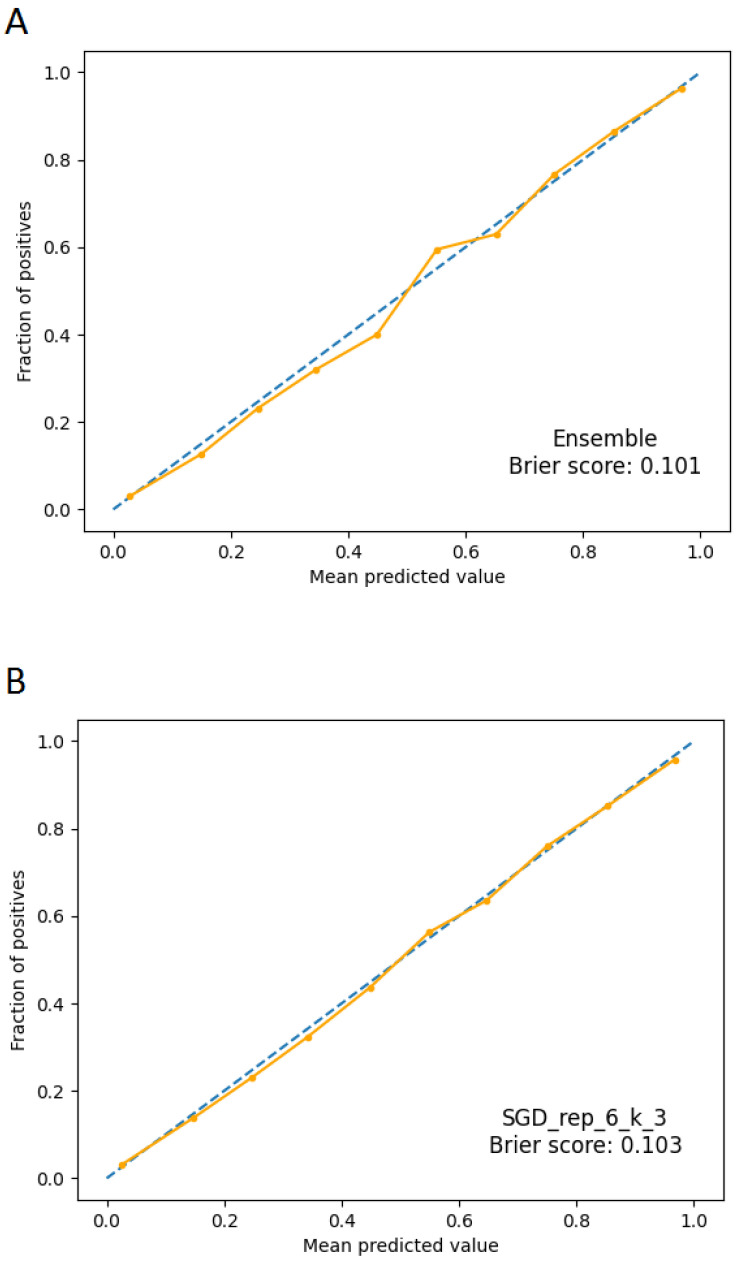
Calibration plots. The dashed line represents a perfectly calibrated ideal model; the continuous line represents the applied model. Panel (**A**): calibration plot for ensemble model. Panel (**B**): calibration plot for SGD model.

It may be preferable when using ML classifiers to have the model estimate probabilities of data belonging to each potential class rather than simple class labels. Having access to probabilities is helpful for giving the responses a more nuanced interpretation or identifying model flaws. If an ML model generates calibrated probabilities, it has been calibrated. In more detail, probabilities are calibrated so that a class forecast made with confidence p is accurate 100 *p% of the time. By using calibrated probabilities, we may take the resulting values and interpret them as representing the model’s confidence. Making a calibration plot is the most typical method for evaluating the model’s calibration. The calibration plot for the ensemble has been calculated on the test set, obtaining the plot shown in Figure 4A. It presents two lines: the dashed one represents a perfectly calibrated ideal model, and the other one indicates the ensemble to be validated. The closer the latter is to the former, the more well-calibrated the model is. To not rely just on the visual data when evaluating the model calibration, the Brier score has also been used; it is essentially the same calculation made for the mean squared error, but it is applied when comparing probability predictions with the actual results of specific events that have been observed. The Brier score ranges from 0 to 1 (the lower the value, the better), with 0 denoting flawless calibration, where the anticipated probability exactly matches the observed probabilities. The value obtained in our case is 0.101 for the ensemble, while it is equal to 0.103 in the case of the SGD neural network (Figure 4B).

## 3. Discussion

Many efforts are oriented towards improvements in diabetes prevention, diagnosis, and care. Applications of AI methods are the most advanced approach based on computational resources. Data obtained by clinical studies should be opportunely integrated within AI approaches, as well as information from investigations at the molecular and cellular levels. As an example, the role of parameters used in our work as features is the object of studies reported in the literature [30,31,32,33,34,35], and novel biomarkers for the evaluation of diabetes and diabetes-related complications could be added in the future, as evidenced by studies on the role of erythrocytes [36].

Since the numerousness of the data is a crucial point in representing a given phenomenon, our work has focused on being able to construct a dataset with large, high-quality data. A well-designed dataset is essential for the success of training and evaluating neural networks, as the quality and representativeness of the data will significantly impact the performance of the network. We used three public datasets to extract data, to introduce heterogeneity into the data. Data extracted were preprocessed to remove data with missing values for the features of interest, obtaining a final dataset of 13,687 individuals, i.e., with a similar number of individuals with and without T2DM. In this way, we obtained a balanced dataset with suitable numerousness. The features were selected for the evidence of relationships to T2DM and for the ease of obtaining them, being measurements of common practice.

We decided not to apply any data augmentation techniques, to preserve the quality of the information, which is fundamental for machine learning algorithms as they search for correlations within the data; all rows with implausible or missing values for at least one characteristic were eliminated. The use of a dataset of at least 13,000 samples represents the first step towards models with performances that increasingly represent their true capabilities on unknown data.

The use of a neural network as a machine learning model was chosen due to its ability to approximate any function with a high degree of precision [37]. These models have been extensively used in the diagnosis of various diseases such as tuberculosis [38], malignant melanomas [39], and neuroblastomas [40]. Furthermore, neural networks have shown the potential in enhancing predictive accuracy when the connections between variables are nonlinear or unknown. Studies have demonstrated that neural networks exhibit superior long-term predictive capabilities in bariatric surgery patients [41] when compared to linear [42] and logistic regression models [43].

Our study suggests that the model applied to the dataset generated can predict the T2DM state with very high performances, based on features chosen by the scientific literature [30,31,32,33,34,35].

The most significant features were blood glucose level, HDL level in the blood, diastolic blood pressure, gender, and weight, while triglycerides, age, BMI, and systolic blood pressure resulted less significant.

The ROC curve is a commonly used method for evaluating the performance of (binary) classification models. It uses a combination of the true positive rate (the percentage of correctly predicted positive examples, defined as recall) and the false positive rate (the percentage of incorrectly predicted negative examples) to obtain a snapshot of classification performance.

By analyzing ROC curves, one assesses the classifier’s ability to discern between, for example, a healthy and a sick population, by calculating the area under the ROC curve (Area Under Curve (AUC)). The AUC value, between 0 and 1, is equivalent to the probability that the result of the classifier applied to an individual randomly drawn from the sick group is higher than that obtained by applying it to an individual randomly drawn from the healthy group.

The higher the area under the ROC curve (AUC), the better the classifier. A classifier with an AUC higher than 0.5 is better than a random classifier. If the AUC is less than 0.5, then there is something wrong with the model. A perfect model would have an AUC of 1. ROC curves are widely used because they are relatively simple to understand, capture more than one aspect of classification (taking into account both false positives and false negatives), and allow for visual and low-effort comparisons of the performance of different types of models. In our study, the calculated ROC AUC value is 0.934. This value suggests a high predictive value for the method developed.

To verify that the heterogeneity of ethnicity does not bias the final results, we performed an analysis for each ethnic group, obtaining very similar results (see Supplementary Material).

As can be seen from Figure 4, the best single neural network (SGD) and the ensemble predictions appear to be calibrated, thus interpretable as probabilities of membership in one class or the other. This is also confirmed by the Brier score, whose extremely low values give us confidence about the accuracy of the predictions in probabilistic terms. The calibration of the models must be checked carefully because faulty calibration might result in bad decisions, and reporting both is crucial for prediction models [44].

## 4. Materials and Methods

### 4.1. Features

A set of features was chosen based on literature evidence [30,31,32,33,34,35], and it consists of glucose level in the blood, measured in mg/dL, triglycerides level in the blood, measured in mg/dL, HDL level in the blood, measured in mg/dL, systolic blood pressure, measured in mm/Hg, diastolic blood pressure, measured in mm/Hg, gender/sex expressed as a binary numerical value, age, expressed in years, weight, measured in kg, and Body Mass Index (BMI), expressed in kg/m^2^. The values of these features, together with the diabetes status, were extracted from the datasets described in the next paragraph.

### 4.2. Datasets

Previous studies have highlighted the availability of datasets from various surveys conducted between 1999 and 2018 by the National Center for Health Statistics (National Health and Nutrition Examination Survey, NHANES) [24] as well as two datasets containing clinical data, MIMIC-III [25] and MIMIC-IV [26].

NHANES 1999–2018 datasets provide a nationally representative sample of adult US citizens, aged 18 years or older, in the range of seven thousand individuals for each year. MIMIC-III is a publicly accessible database that contains anonymized health-related information on more than 40,000 patients who received ICU care at Beth Israel Deaconess Medical Center between 2001 and 2012. MIMIC-IV is an upgrade to MIMIC-III that adds modern data and enhances many elements of the previous version.

Each entry within the entire NHANES dataset has a key, called SEQN, which serves as the identifier of the subject to which the data refers.

Since data within the NHANES dataset are distributed over several datasets, the data collection process required an initial phase of searching for the feature vector data and a subsequent phase of merging these, for which Python 3.8.11 together with the Pandas 1.2.4 library was used.

Features such as glucose and triglycerides are distributed in different datasets. For both, the following data retrieval and merging procedure was performed; the different datasets with the feature of interest were downloaded, and then, based on the values in the SEQN column, the rows in which this value was identical were merged, and those in which it was not were merged into a single table. In particular, for the rows that were merged, those in which all had a value Nan (Not a Number) were eliminated. If, on the other hand, the set of rows with the same identifier had at least one non-Nan value, the first one in order of reading was taken.

The remaining features of interest were each located in a single dataset. They were then extracted and merged into a single table based on the SEQN key. This process was repeated iteratively for each dataset from the year 1999 to 2018 and then merged into a single table, based on the SEQN key, for all datasets. At the end of the process, a partial dataset of 48,067 examples was obtained, of which only 4415 had type 2 diabetes.

Both MIMIC datasets (i.e., III and IV) have a size, in terms of rows corresponding to distinct patients, of more than 40,000 examples. The data retrieval methods were the same. Since the information was spread across several tables, the approach taken was to retrieve, based on a key identifying a particular admission of a given patient, each characteristic individually and then combine them into a single table. Some features such as age, sex, HDL, and triglycerides were directly accessible from identification codes. Others, however, required some additional steps before being retrieved: BMI was not present in the data and was therefore calculated from weight (w) and height (h) using the formula: BMI = w/h^2^.

All patients with forms of diabetes other than type 2 were excluded from the selection. To have fasting glucose values, the values retrieved corresponded to analyses performed no later than ten o’clock in the morning, assuming the patient had fasted for at least eight hours. At the end of this phase, 2997 patients were extracted from MIMIC-III, and only 1576 from MIMIC-IV, as the largest subset of patients containing all the characteristics of our interest. The possibility of filling in the missing gaps with aggregation functions on the data present was discarded, since the gaps were very large; in fact, for some features, up to 70% of the rows were missing that particular information.

### 4.3. Preprocessing

At this stage, the retrieved data were skimmed, appropriately coded, and finally merged into a single dataset.

Rows that had at least one implausible value for any of the features or had it as null (i.e., it does not have a value) were removed. Implausible values were considered on the following criteria: (i) diastolic blood pressure values exceeding 220 mm/Hg; (ii) BMI values greater than 100 kg/m^2^; (iii) age values greater than 100; (iv) Triglyceride values greater than 900 (mg/dL). The rows removed due to implausible values were 95. Outliers were not eliminated.

At this point, the rows from the three datasets were concatenated and, as the number of non-diabetics was enormously larger than that of diabetics, an under-sampling of the first class was carried out, resulting in a table of approximately 13,000 rows, with a balanced number of non-diabetics and diabetics.

All data underwent a standardization process, as the vector characteristics of interest have different units.

### 4.4. Neural Networks: Model’s Architecture

**Figure 5:**
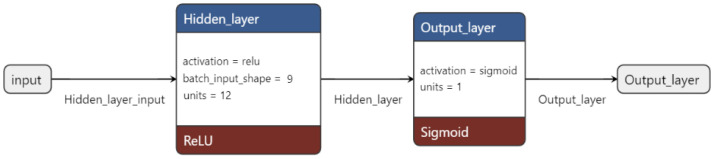
The diagram illustrates the architecture of the neural network. The input layer is represented by the gray square, with the hidden and output layers depicted in blue. The activation functions used are indicated in red. The number of units per layer is specified within each respective square.

The neural network was designed as a shallow fully connected architecture, featuring a single hidden layer. This architecture is characterized by the property that each neuron in each layer receives connections from all neurons in the previous layer, except for the input layer. In Figure 5, we report a schematic draw of the architecture of the neural network.

### 4.5. Validation

Once the dataset was balanced, it was divided into two subsets: a training set of size 80% of the total, and a test set with the remaining 20%. The former was initially used for the optimal search for the number of nodes in the hidden layer of the neural network. In fact, it was further partitioned, according to an 80:20 ratio, into an additional training set and a validation set. A grid search was performed on these two in a search space, understood as the number of nodes, equal to the interval [5,15]. For the search of the best optimization algorithm, the first training set was used, on which a k-fold cross-validation with k = 9 was applied, with experiments repeated 11 times. The algorithms considered were Adam, SGD, RSM-prop, and Levenberg–Marquardt (LM). An ensemble of 99 models was created for each of the optimizers, the performance of which was evaluated on the test set. The ROC curve and resulting AUC value were also calculated on the latter.

### 4.6. Calibration Plots

It is possible for statistical models to produce predictions that are uncalibrated, which means that the anticipated values lack the nominal coverage probability. The probabilities of occurrence for popular species in machine learning categorization make this the simplest to see. Before evaluating or averaging uncalibrated probability predictions in a probabilistic manner, they should first be calibrated [45]. It is shown that a model’s calibration, or how closely calculated risks match observed event rates, has an impact on clinical utility [46].

## 5. Conclusions

The research demonstrates the potential of binary classifiers trained from scratch to generalize the onset of diabetes in nonlinear relationships with specific patient measurements. The ablation study revealed that an ensemble of binary classifiers with a shallow architecture optimized using the Adam algorithm attained a satisfactory level of accuracy (approximately 86% on the test set) and an ROC AUC value of 0.934.

This neural network-based approach may provide accurate information for personalized medicine, making it a valuable resource for decision making.

Further studies incorporating multiple information of the same patient over time could lead to the development of an advanced model for disease prevention. This would be possible by identifying patterns, such as context patterns in the trends of the measurements, using advanced neural networks such as Long-Short-Term-Memory models.

## Figures and Tables

**Table 1:** Data statistics before the preprocessing.

Feature	Count	Mean	Standard Deviation	Minimum Value	Maximum Value
Gender/Sex ^a^	52,640	1.512	0.499	1.0	2.0
Age (years)	52,640	43.764	24 336	12.0	300.0
Diabetes ^b^	52,640	0.130	0.337	0.0	1.0
HDL-cholesterol (mg/dL)	52,640	52.112	15.535	3.0	226.0
Glucose (mg/dL)	52,640	103.255	36.607	21.0	683.0
Systolic: Blood pres (mm/Hg)	52,640	121.440	18.820	51.0	270.0
Diastolic: Blood pres (mm/Hg)	52,640	68.041	12.938	21.9	676.1
Triglycerides (mg/dL)	52,640	139.093	122.210	9.0	6057.0
Weight (kg)	52,640	78.153	21.506	25.1	371.0
Body Mass Index (kg/m^2^)	52,640	33.061	948.424	3.24	215.7

^a^ The gender/sex is reported with a value (1 for male, 2 for female). ^b^ The pathological status is assigned.

**Table 2:** Data statistics after the preprocessing.

Feature	Count	Mean	Standard Deviation	Minimum Value	Maximum Value
Gender/Sex ^a^	13,687	1.543	0.498	1.0	2.0
Age (years)	13,687	51.947	21.179	12.0	99.0
Diabetes ^b^	13,687	0.498	0.500	0.0	1.0
HDL-cholesterol (mg/dL)	13,687	49.914	15.607	3.0	158.0
Glucose (mg/dL)	13,687	124.347	56.437	21.0	649.0
Systolic: Blood pres (mm/Hg)	13,687	124.506	19.873	51.0	242.0
Diastolic: Blood pres (mm/Hg)	13,687	67.498	12.430	21.9	202.3
Triglycerides (mg/dL)	13,687	152.276	107.965	12.0	896.0
Weight (kg)	13,687	82.788	22.865	27.8	273.0
Body Mass Index (kg/m^2^)	13,687	29.456	7.313	3.2	97.4

^a^ The gender/sex is reported with a value (1 for male, 2 for female). ^b^ The pathological status is assigned.

**Table 3:** Average accuracy values for each experiment (the higher the best).

Hidden Nodes	Accuracy
12	0.838
13	0.838
14	0.837
15	0.837
11	0.837
10	0.836
7	0.835
5	0.835
9	0.835
6	0.834

**Table 4:** Average accuracy values for each optimizer (the higher the best).

Optimizer	Mean (Accuracy)	Standard Deviation (Accuracy)
ADAM	0.855	0.008
SGD	0.853	0.009
RMSPROP	0.852	0.009
LM	0.835	0.049

**Table 5:** Average accuracy values for each experiment on ensemble models (the higher the best).

Model	Accuracy
SGD	0.862
RMSPROP	0.861
ADAM	0.858
LM	0.840

## Data Availability

Data used in this study were from public datasets (see Section 4).

## Supplementary Materials

The following supporting information can be downloaded at: https://www.mdpi.com/article/10.3390/ijms24076775/s1.
