# Supplementary material for: Machine Learning as a Support for the Diagnosis of Type 2 Diabetes
Source: Int J Mol Sci. 2023 Apr 5;24(7):6775. doi: 10.3390/ijms24076775 (PMC10095542; doi:10.3390/ijms24076775)

Supplementary material

Machine learning as a support for the diagnosis of type 2 diabetes

Antonio Agliata [1,2], Deborah Giordano [3], Francesco Bardozzo [1], Salvatore Bottiglieri [2], Angelo Facchiano [3,*] and Roberto Tagliaferri [1]

[1] Dipartimento di Scienze Aziendali, Management and Innovation Systems, Università degli Studi di Salerno, 84084 Fisciano, Italy – aagliata@unisa.it, fbardozzo@unisa.it, robtag@unisa.it
[2] BC Soft, Centro Direzionale, Via Taddeo da Sessa Isola F10, 80143 Napoli, Italy – antonio.agliata@bcsoft.net, salvatore.bottiglieri@bcsoft.net
[3] National Research Council, Institute of Food Science. Via Roma 64, 83100 Avellino – deborah.giordano@isa.cnr.it, angelo.facchiano@isa.cnr.it
* Correspondence: angelo.facchiano@isa.cnr.it

In the following pages, ROC curves and AUC values are reported for the analysis on each ethnical group.

Ethnic group: WHITE

ROC AUC=0.930

Number of samples: 1239

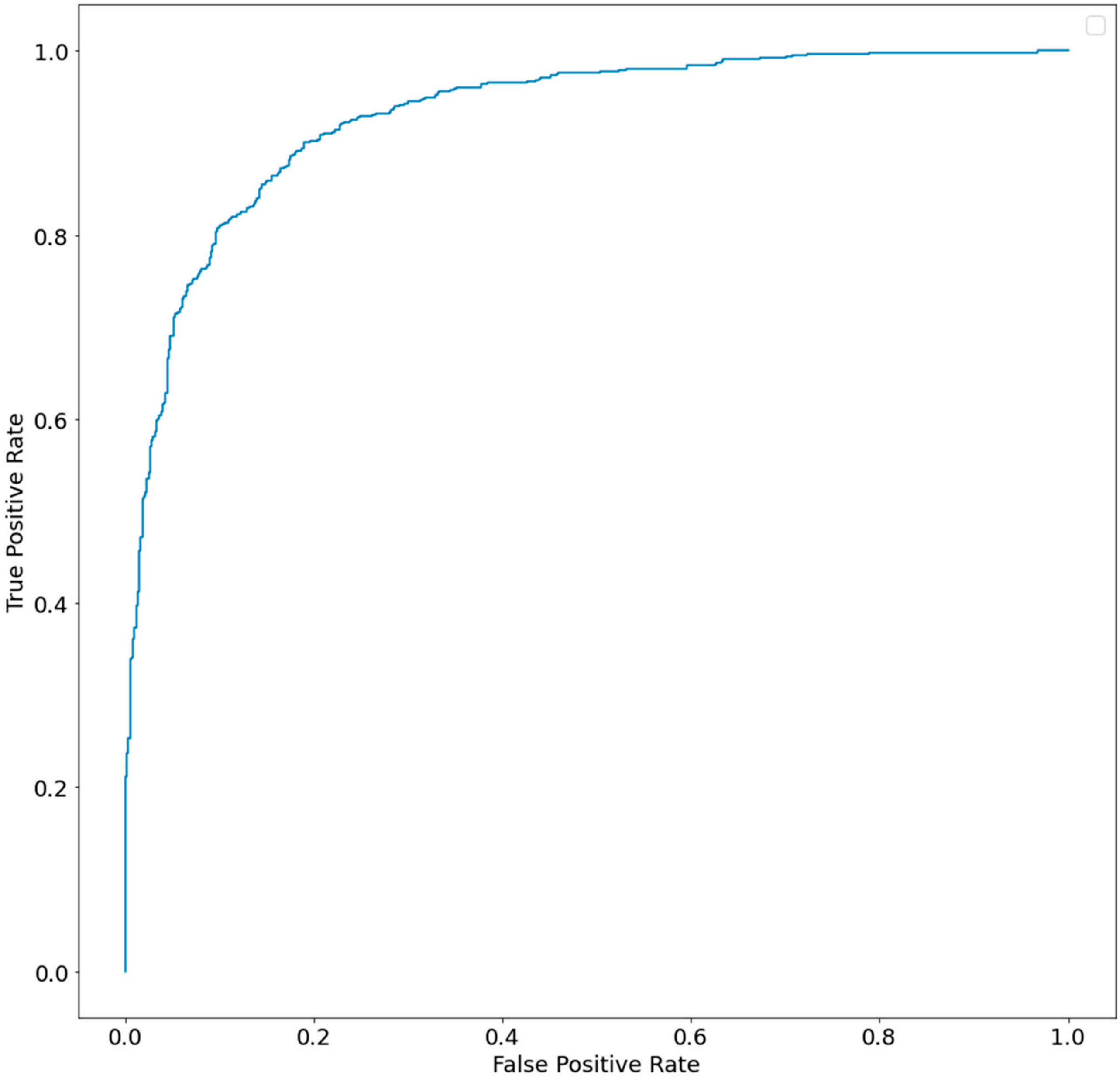

Ethnic group: BLACK:

ROC AUC=0.901

Number of samples: 530

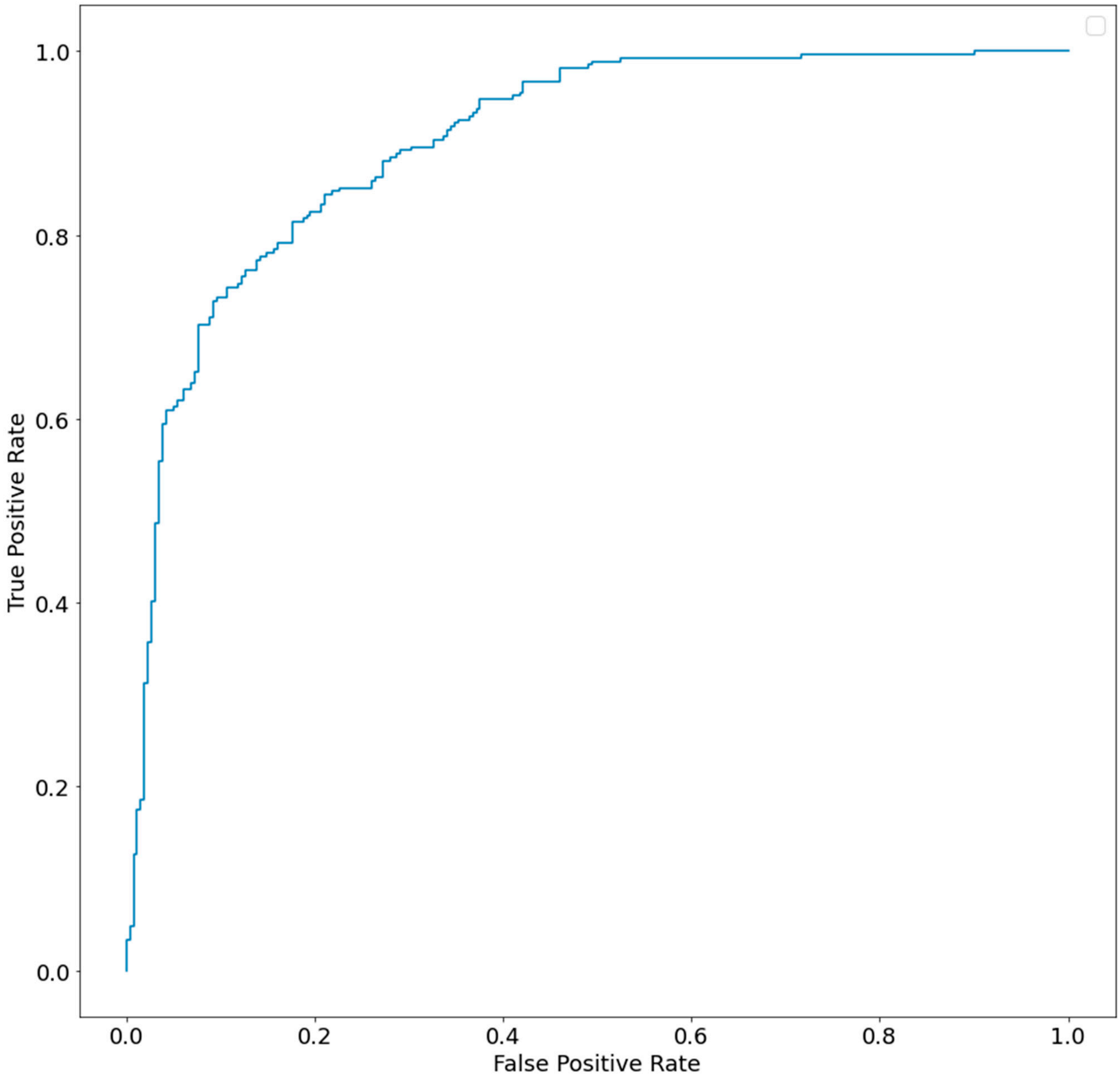

Ethnic group: ASIAN

ROC AUC=0.937

Number of samples: 123

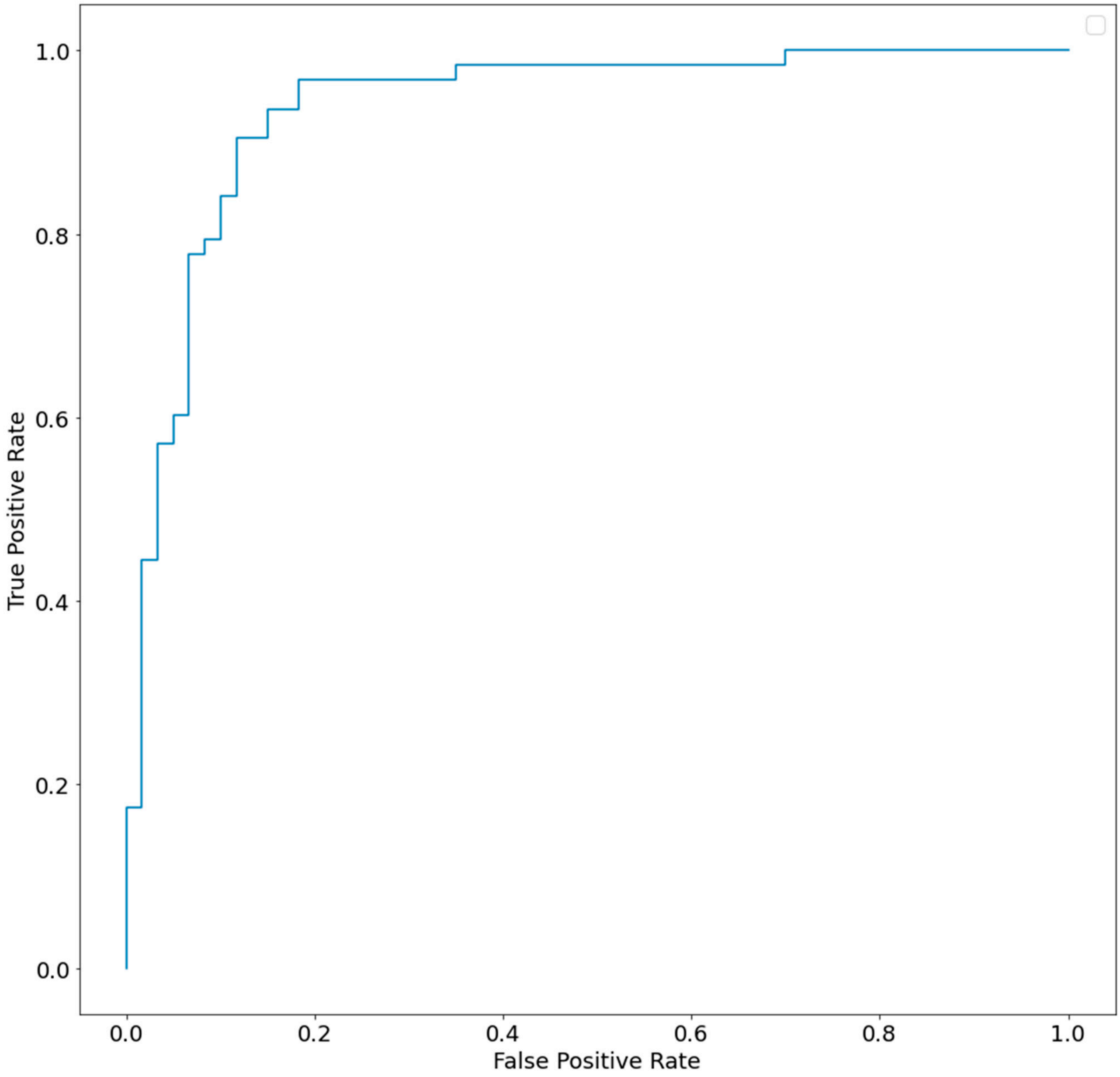

Ethnic group: HISPANIC

ROC AUC=0.934

Number of samples: 670

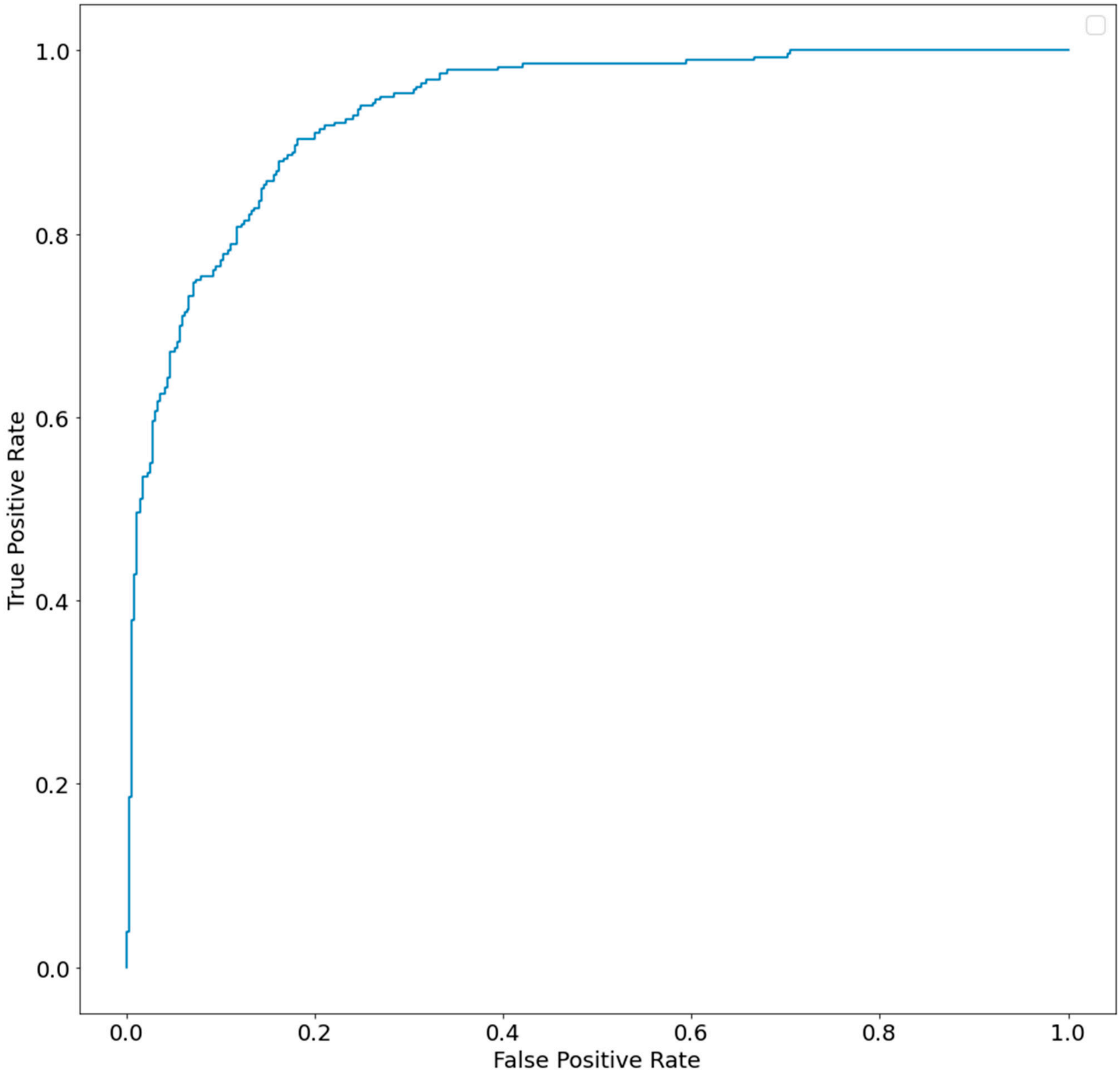

Ethnic group: OTHERS

ROC AUC=0.971

Number of samples: 176

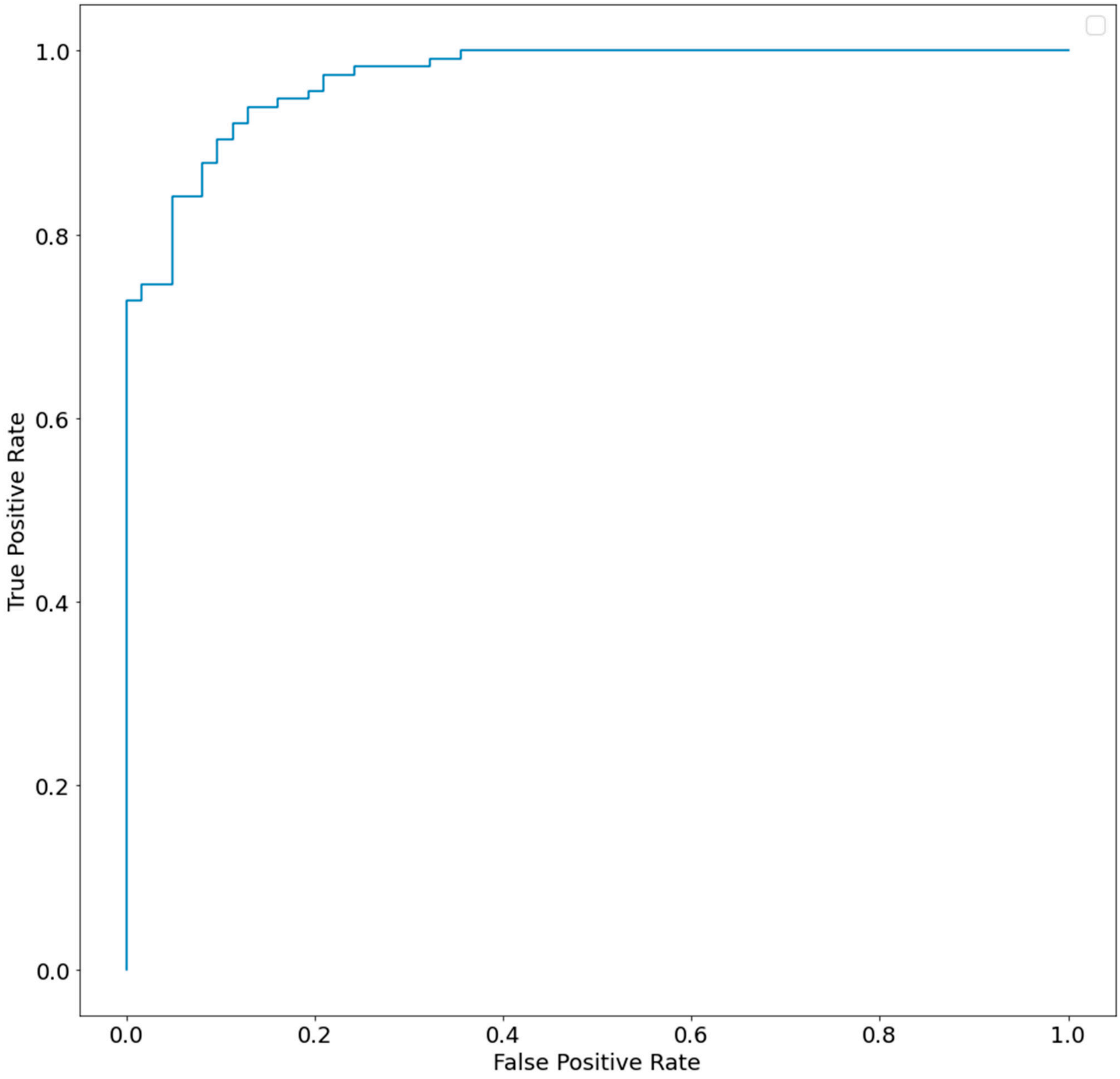

Supplement: Supplementary file 1 [file ijms-24-06775-s001.zip › ijms-2253820-supplementary.pdf]
